# The thin line between biochemistry and structure where myosin super relaxed state lies

**DOI:** 10.1007/s12551-026-01433-y

**Published:** 2026-04-15

**Authors:** Leonardo Nogara, Carlo Reggiani

**Affiliations:** 1 Department of Biomedical Sciences, University of Padova, Padua, Italy; 2 Department of Pharmaceutical and Pharmacological Sciences, University of Padua, Padua, Italy

**Keywords:** Skeletal muscle myosin, Super Relaxed State, Roger Cooke

## Abstract

This short review acknowledges the scientific contribution of Roger Cooke and colleagues, who first introduced the idea of two different biochemical states in resting skeletal muscle thick filament. In a seminal paper published in 2010, the state defined as myosin “super relaxed state” (SRX) has been proposed as the biochemical counterpart of the structural motif named interacting head motif (IHM). This connection has been challenged and revised multiple times, leading to misunderstandings and improper assumptions. In this review, we discuss the recent history of the structure–biochemistry relationship associated with resting myosin thick filament, its current interpretation, and how this is possibly related to future myosin resting state studies.

## Preface

Myosin structure has fascinated scientists for decades. The physiological relevance of a protein involved in uncountable functions, in combination with the challenging mechanical description of a molecular motor, has pushed structural research since its early days. While the coupling of nucleotide hydrolysis to the force development of the power stroke attracted most interest at first, we have been lately experiencing great attention to the motor resting state.

The closed conformation of myosin motors has been detected in many vertebrates and nonvertebrate species, suggesting the possibility of the evolutionary refinement of the motor structure happening simultaneously with the intrinsic energy sparing mechanism (Lee et al. 2018). While early descriptions reported on mammalian smooth muscle myosin (Wendt et al. 2001; Liu et al. 2003), more recent research driven by translational relevance focused instead on striated muscles. The greatest interest has been attracted by the important role of resting myosin states in cardiomyopathies and on the pharmacological modulation of cardiac contractility. In skeletal muscles, myosin resting state studies pivot around their implication in basal metabolism, with potential influence over metabolic conditions.

Considering the recent additions of myosin SRX-DRX dynamics in the field of muscle research and the multiple yet undiscovered scenarios related to its alteration, this review will essentially cover class II myosins in mammalian skeletal muscles. We recognize the significant differences between cardiomyocytes and skeletal myofibers regarding their myosin isoforms and sarcomeric protein complements. This work aims to clarify the relationship between the biochemistry of the Super Relaxed state and the structural orientation of the myosin head, focusing primarily on skeletal muscle. As research on skeletal muscle is less extensive than that on cardiac tissue, we took the liberty of extrapolating certain findings from the latter. Our goal is to leverage the analogies between these two tissues to present our interpretation of the subject, rather than to detail their specific physiological differences. Consequently, this discussion intentionally excludes references to mutations responsible for cardiomyopathies, as well as the relationships between resting skeletal myosin status and factors such as physical activity levels, hormonal status, diabetes, and obesity.

## Introduction

**Fig. 1:**
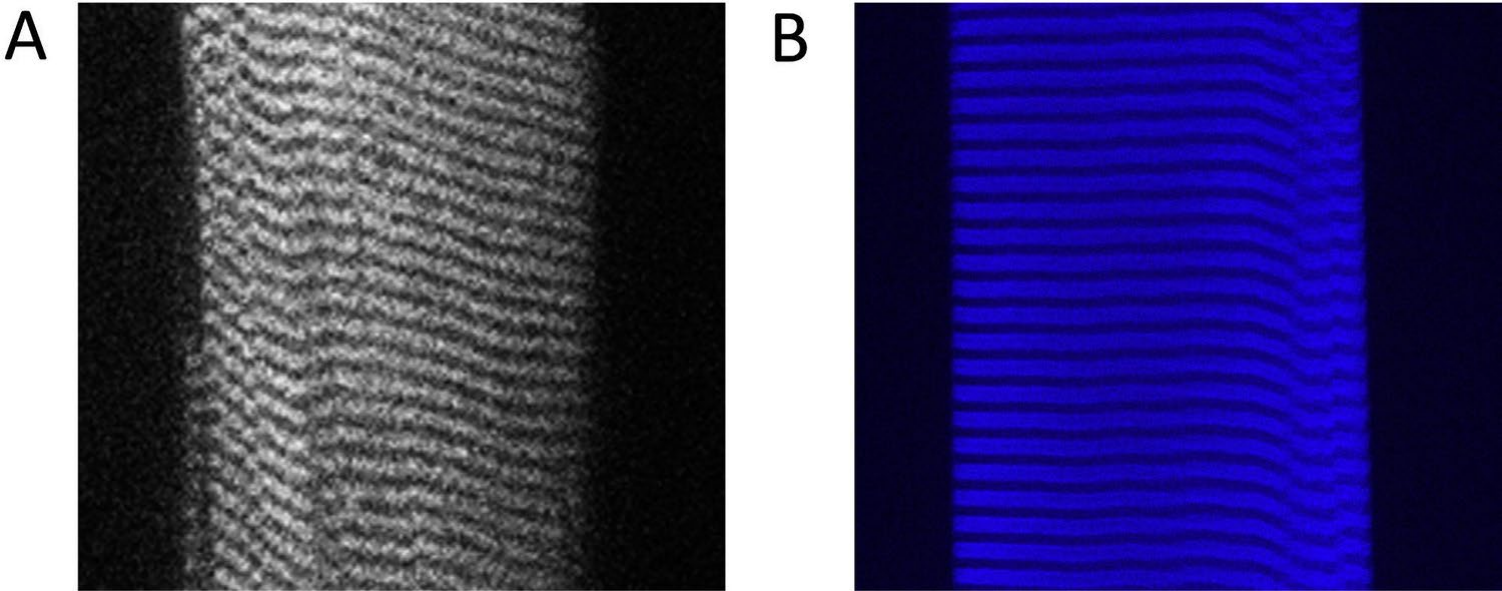
Side-by-side comparison of the confocal image of mantATP on skinned muscle fibers as in the avant-garde original research published by the Cooke laboratory (Stewart et al. 2010) (**A**) and, 15 years later, the analogous picture as in Montesel et al. (Montesel et al. 2025) (**B**). Permission for reproduction original content granted by PNAS executive editor.

The energy consumption of resting skeletal muscle tissue is very low (Elia 1992), but due to the large mass of skeletal muscles, its contribution covers approximately 30% of basal metabolism (Zurlo et al. 1990). The metabolic activity of skeletal muscle at rest, although very low, is relevant for thermogenesis, for regulation of blood sugar levels, and more in general to the anabolic–catabolic balance. The determination of ATP consumption of purified myosin, however, is much higher than expected on the basis of resting muscle metabolism (Kushmerick and Paul 1976). Such discrepancy was reported back in the ‘70s (Ferenczi et al. 1978), but it was precisely defined only 20 years later by Roger Cooke and coworkers (Myburgh et al. 1995), who developed the mantATP chase experiments. Importantly, the fluorescence decay of mantATP, when interpolated with a bi-exponential function, suggested the existence of two populations of myosin motors with distinct turnover rates (Stewart et al. 2010). In Fig. 1, we report the confocal fluorescence mantATP visualization of the original Stewart et al. article and, fast-forwarding 15 years, the analogue representation obtained in our lab (Montesel et al. 2025). According to Roger Cooke’s view, along the thick filaments of relaxed muscle fibers, myosins motors are present in two states, called super relaxed state (SRX) and disordered relaxed state (DRX), respectively (Stewart et al. 2010). This discovery will become very relevant for its influence over cardiac muscle contractility, which was anticipated by Cooke (Hooijman et al. 2011, McNamara et al. 2014) and opened the field of pharmacological control of resting myosin heads to modulate cardiac output. In skeletal muscle, in addition to contractility, it gained interest for its potential control over tissue basal metabolism (Cooke 2011; Wilson et al. 2021).

The idea that resting myosin heads were ordered along the backbone of the thick filament in striated muscles was proposed as early as the ‘60s, when pioneering X-ray diffraction patterns suggested that in relaxed skeletal muscle a consistent mass was arranged in helicoidal order on the filament surface (Huxley et al. 1965; Huxley 2004). The first molecular model of a myosin dimer structural arrangement in the closed conformation only arrived in the 2000 s on tarantula muscle samples (Woodhead et al. 2005; Alamo et al. 2008). In the frame of the dogma that function and structure are related at the molecular level, that asymmetric structure, characterized by multiple protein–protein interactions, reasonably became the conformational representation of the lower turnover rate state or SRX.

Since the time of those pioneering studies, the existence of two or more states of myosin in resting skeletal and cardiac muscles has collected robust evidence both at structural and biochemical levels. However, the connection between structure and function has proved to be more complex than expected.

## Evidence in favor of two or more myosin states in resting fibers

The *model* of myosin obtained by *cryo-EM* in the resting thick filament of tarantula striated muscles (Woodhead et al. 2005) gave the first portrait of the possible head arrangement that could generate the resting X-ray patterns since Huxley’s first observations. Multiple key features were described by this model, a main one being myosin heads’ asymmetry within the dimer. Two motors with identical amino acid sequences and the same auxiliary light chains, that are notoriously nonprocessive during contraction, suddenly join in this resting conformation in which one is named “free head,” FH, and the other is “blocked head,” BH. The model has been named the interacting head motif (IHM) and has been identified in virtually all class II myosin isoforms although with distinct features (Padrón et al. 2022 Five main intra-dimer interactions have been revealed by the atomic resolution CryoEM structure of cardiac HMM with 15 heptades coiled-coil: head-head, blocked head-coiled-coil, RLC(BH)-RLC(FH), ELC(BH)-RLC, ELC(FH)-RLC. In the frame of the hypothesis that cooperativity drives OFF to ON myosin recruitment, the model highlighted intermolecular interactions among heads (Grinzato et al 2023). 

The resolution of the IHM structure in the frame of a supra-molecular structure has been improved down to the nearly atomic level, once again with the cardiac murine (Tamborrini et al. 2023) and cardiac human (Dutta et al. 2023) thick filament reaching outstanding, unprecedented details and documenting novel interactions with titin and myosin binding protein C (MyBP-C). The latter proteins play possibly a regulatory function but are not directly required for IHM formation. These new models overtake the “dimer” conception, introducing the concept of head crowns and displaying additional interactions all along myosin tail, as was never possible before. Along the C zone of the thick filaments, distinct types of IHM can be detected. While some crowns appear stabilized by cMyBP-C and likely correspond to the SRX state, other crowns are more mobile and likely correspond to the DRX (Dutta et al. 2023). Despite being limited to the C zone, and stabilized by mavacamten, these models are the best representation ever obtained of a myosin heads’ arrangement. A similar level of detail has not yet been reached for skeletal muscle thick filament.

The helical ordering of the myosin heads at rest arising from the IHM structures arranged along the thick filament has been linked with classical observations with X-ray diffraction. A radial mass transfer from the thick filaments during contraction was observed 50 years ago and interpreted as the loss of an arrangement of the myosin head domains on the surface of the thick filaments in resting muscle (OFF) (Haselgrove and Huxley 1973). The OFF structure has a characteristic X-ray pattern, where the three-stranded pseudo-helical arrangement of the myosin motors on the filament surface produces a series of layer-line reflections that are orders of a fundamental axial periodicity of about 43 nm (ML1), with the axial repeat of the motors (M3) of 14.34 nm and a periodic mass distribution in the filament backbone with a periodicity (*M6*) of 7.17 nm. As shown by Koubassova et al. (Koubassova et al. 2025), such pattern can be predicted based on a filament model with IHM on the surface. The OFF pattern is lost when activation occurs and sufficient load is applied on the filaments (Linari et al. 2015).

While change in intensity of M3 and M6 is often adopted as a reference of mass transfer associated with the OFF/ON transition, it must be considered that it can only be sensitive to changes in average mass distribution, making it unlikely able  to estimate the proportion of constitutively disordered heads.

Structural OFF/ON transitions in the thick filament could be detected by recording polarized fluorescence from *bifunctional rhodamine* probes at four α-helices of the myosin regulatory light chain (RLC). This approach reveals the orientation of the C-lobe of the RLC in permeabilized muscle fibers (Fusi et al. 2015). At 25 °C and above, three preferred orientations of the myosin RLC were present, two with long axis roughly parallel and one roughly perpendicular to thick filament axis. In relaxed myofibers, the majority of the myosin heads are oriented parallel to the filament axis and, thus, reminiscent of those of the so-called blocked and free heads of IHM conformations. Only a minority shows a distinct conformation with their RLC regions roughly perpendicular to the filament axis. All those three orientations disappear during active contraction. This approach gives very relevant, dynamic information of myosin lever arm orientation, but it could be somehow limited by the fact that RLC exchange efficiency reaches about 50% at most. It has not been established yet whether this ratio would be due to a preferential exchange of the free or blocked head, or if it depends on thick filament myosins subpopulations (e.g., proximal zone, C-zone or distal zone, D-zone).

The analysis of the *biochemical counterpart* of the distinct myosin relaxed states (SRX and DRX) has also evolved from the original bi-exponential fitting to interpolate the curve of mantATP chase proposed by Roger Cooke and coworkers (Stewart et al. 2010). The mantATP chase experiments have been repeated on a variety of preparations, showing two states with distinct ATPase rates not only in individual myofibers but also in reconstituted thick filaments, HMM preparations i.e two heads with S2 segment with 8-15 heptades and, surprisingly, even in single head (S1) preparations (Anderson et al. 2018). Stopped flow methods were also applied to follow the decay curve in mant-ATP chase experiments (Walklate et al. 2022). An original and appealing alternative has been designed by David Warshaw and coworkers (Nelson et al 2020). A super-resolution microscope allowed imaging of single molecules of fluorescent BODIPY-ATP dispersed in a high concentration of nonlabeled ATP during hydrolysis events in relaxed myofibrils of rat soleus. A similar experiment was also carried out in myofibrils of rabbit psoas to highlight the effect of protein kinase A (PKA) on myosin resting ATP turnover (Pilagov et al. 2022). This approach allowed not only the detection of the kinetic parameters, revealing once more two myosin states with distinct ATPase rate, but also the spatial localization along the thick filaments (Rasicci et al. 2022).

## Factors altering myosin resting structure and biochemistry

The most established physiological modulator of myosin SRX stability is phosphorylation due to myosin light chain kinase (MYLK2 in skeletal muscle), as already reported in the Cooke lab original paper (Stewart et al. 2010). In the following years, many other factors such as ionic strength, temperature, and a number of small molecules (mavacamten, Omecamtiv, piperine, deoxy-ATP, etc.) were shown to modulate the biochemistry of SRX/DRX balance and its potential structural counterpart. On this ground, the function–structure link has been subjected to several validation experiments, almost exclusively on myosin fragments or ex vivo preparations. The identification of SRX with IHM could be supported by parallel changes when purified myosin motors or native thick filaments or myofibers are exposed to certain interventions. The outcomes of these interventions might differ based on the myosin heavy chain isoform considered, which affects intrinsic SRX stability, or on the specific sensitivity for a molecule (e.g. piperine).

*High salt* concentrations reduce IHM configuration and decrease the amplitude of the slow phase of ATP turnover (SRX), an effect partially counteracted by mavacamten (Lee et al. 2018; Rohde et al. 2018). However, evidence against the modulation of IHM structure by ionic strength increase has been reported (Chu et al. 2021). SRX/DRX ratio increases with *temperature* in the range 12–35 °C (Stewart et al. 2010), 15–30 °C (Nogara et al. 2016a), and 5–30 °C (Walklate et al. 2022). Accordingly, the order parameter P2 of polarized fluorescence of bifunctional rhodamine probes increases with temperature (range 5–35 °C) indicating an orientation more parallel to the thick filament axis (Fusi et al. 2015). The low angle X-ray diffraction shows that low temperatures favor motor disordered conformation, while high temperatures favor the ordered (closed) OFF conformation: range 15–25 °C (Xu et al. 2003), 15–30 °C (Nogara et al. 2016a), and 10–35 °C (Caremani et al. 2019). Importantly, a temperature reduction at 10 °C or below shifts the heads into a different, locked OFF state (Caremani et al. 2019). As first shown by Anderson et al. (Anderson et al. 2018), *mavacamten* stabilizes the SRX state, increases SRX/DRX, the folded or closed, IHM-like, conformation of cardiac HMM and the myosin-based helical layer line reflections in relaxed cardiac fiber by low-angle X-ray diffraction. The observations on Mavacamten supporting the identification SRX-IHM were questioned by Chu et al. (Chu et al. 2021), who pointed out that the Mavacamten effect on SRX-DRX balance is linear with the dose, while the structural effect is nonlinear, defining the IHM sufficient but not necessary to produce the SRX kinetic state. The effect of *piperine* in lowering the SRX/DRX ratio was discovered by Nogara et al. in a screening of thousands of compounds (Nogara et al. 2016a). The structural destabilization was then confirmed as the molecule induces an OFF to ON shift of the X-ray diffraction pattern by Jani et al. (Jani et al. 2024). The latter study, however, also pointed to a function–structure decoupling as the shifts from OFF to ON in porcine cardiac specimens induced by piperine as well as omecamtiv was not accompanied by any variation in SRX-DRX balance as determined by mantATP chase. It is worth to note that piperine has been found to increase ATP hydrolysis only on fast myosin isoforms (Nogara et al. 2016a), while porcine myocardium expresses slow/beta myosin isoforms. An interesting observation is that mavacamten and omecamtiv, despite favoring two different configurations, bind myosin in the same pocket (Auguin et al. 2024), possibly highlighting a pivotal key location to shift the structural equilibrium.

Doubts on the IHM-SRX identification arise from the observation of two phases of fluorescence decline in S1 fragment (Anderson et al. 2018), where only one head is present and, thus, the IHM cannot exist. The unexpected behavior might be explained by the presence of a fraction of S1 fragments blocked in pre-power stroke with inhibited Pi release, even without the need for interaction between the two heads. This could imply that in IHM not only the BH but also the FH might share the blocked Pi release condition (Anderson et al. 2018).

A relevant objection to the identification of IHM as the counterpart of SRX was forwarded by Walklate et al. (Walklate et al. 2022). In the presence of IHM, the slow release of Pi and the completion of the ATPase cycle could be due to either the SRX-DRX transition or to slow direct release of Pi from the blocked head. Comparing the rate constants allows us to distinguish between the two alternatives.

(i) If k_cat,DRX_ >> k_exit,SRX_, then the net turnover rate is given by the rate of exit from the SRX state (k_exit SRX_)).
(ii) If k_cat,DRX_ << k_exit,SRX_, then net turnover rate is given by k_cat,DRX_.

More specifically, if the ATP turnover rate of SRX is 0.005 s^−1^, the SRX → DRX transition cannot be faster; otherwise, ATP turnover occurs through a transition in DRX, which has a turnover rate 10 times higher and thus completes the cycle with the release of Pi and ADP.

The comparison between the rate of ATP turnover by myosin in SRX state and the rate of transition SRX → DRX was discussed also by Cail et al. (Cail et al. 2025), who, despite working with a single exponential, noted that the turnover rate of S1 is higher than that of HMM. From this, they conclude that HMM introduces a brake on the release of Pi, and this is likely due to the presence of SRX and IHM. The absence of a discrete slow kinetic phase (often identified as a second exponential in other literature) is explained by the fact that the SRX-DRX transition is much faster than the subsequent product release; this rapid exchange prevents the populations from appearing as distinct kinetic components and instead results in a single observed rate.

An interesting objection to the use of mant-ATP fluorescence decay was raised by Mohran et al. (Mohran et al. 2024) based on the asymmetry between the kinetics of SRX formation and the kinetics of ATP turnover. Upon addition of mant-ATP to a permeabilized fiber in rigor, the fiber transitions from rigor to a relaxed state with a high fraction of SRX occurring in less than 1 second. Thus, the rate of entry into SRX is on the order of 10 s^−1^, and the rate of exit to DRX must be faster than the ATP turnover rate, likely on the order of 0.05 s^−1^.

Mohran's paper (Mohran et al. 2024) also showed that interpolation with a single exponential is sufficient to describe the decline in mantATP fluorescence with a rate corresponding to that of the steady-state ATPase. The criticism of the two-exponential interpolation to identify the SRX and DRX components was also supported  by Cail et al. (Cail et al. 2025), who showed that taking into account FRET and correcting for photobleaching eliminates the slow component in the decay of mantATP fluorescence. Interestingly, the residual monoexponential shows a rate (or a time constant) influenced by mavacamten, as expected.

Beside photobleaching and possible FRET, further critical aspects of SRX analysis based on interpolation with two exponentials are represented by slow diffusion and nonspecific interactions with other ATPases (SERCA, sodium-potassium pump, etc.). These aspects have been examined and discussed in several studies as early as the initial SRX paper by Stewart at al. (Stewart et al. 2010), by  Anderson et al. (Anderson et al. 2018) and, recently, by Montesel et al. (Montesel et al. 2025). The latter study has quantified the impact of nonspecific interactions by performing the mantATP chase experiment in “ghosted fibers” where 95% of myosin had been removed by high KCl solutions. The rate constant of fluorescence decay of “ghosted fibers” was comparable to that of the fast DRX component of classical chase experiments and attributed to nonspecific binding and diffusion. This allowed a correction of the parameters obtained by the decaying curve fitting (Montesel et al. 2025).

## Discussion and conclusions: how to re-interpret the function–structure relation

All these critical observations taken together cast serious doubts about the direct identification of IHM as a necessary structural counterpart of SRX. It is, however, beyond doubt that the ATP hydrolysis rate of resting muscle fibers is lower than that of purified myosin (Ferenczi et al. 1978) as underlined by Roger Cooke (Myburgh et al. 1995). Moreover, not only the mant-ATP chase experiments but also the determination of the turnover rates of single ATP hydrolysis events using BODIPY ATP or Cy3 ATP points clearly to the presence of two distinct populations with 5 times or more difference in ATPase rate (Nelson et al. 2020; Pilagov et al. 2022). In this frame, the correspondence of SRX on the functional side and IHM on the structural side still seems to provide a reasonable explanation. The relation between the two cannot, however, be so direct as initially thought. The existence of two populations of resting motors with different enzymatic activity and possibly different kinetics of transition to actin interaction remains the main contribution of the original 2010 paper by Roger Cooke, with rich implications for basic as well as translational science.

If we assume that the turnover rate of myosin ATPase at rest, that is, without interaction with the thin filament, is limited by Pi release in a pre-power stroke state (PPS), then the different rates observed in solution of single myosin heads, S1, suggest that the existence of two different states, with different rates as proposed by Anderson and coworkers (Anderson et al. 2018).

If we then move to a two-head configuration of the myosin molecules, as in HMM or even in more structurally organized molecular arrangements as filaments, myofibrils, or fibers, the PPS state can be further modulated by a variety of molecular interactions. The HMM configuration implies the slow turnover rate of the BH is determined by the interaction with the FH and likely also with the S2 backbone (see Grinzato et al. 2023). Such intradimer cooperation can develop and be modulated by MyBP-C or titin and a variety of small molecules. The different levels of cooperativity inside dimers, among dimers, and along the filaments and their impact on fluorescence decay curves have been recently explored (Montesel et al. 2025), based on a mathematical model; the inclusion of structural constraints allows the generation of a multi-exponential nucleotide release which nicely fits with experimental results. 

In native filaments, myofibrils, and fibers, more protein–protein interfaces can contribute to the establishment and maintenance of the close conformation, favoring the slow nucleotide release. As shown, an alteration of these contacts as small as a regulatory light chain point mutation favors the disordered state (Nogara et al. 2016b) possibly taking advantage of filament cooperativity. More recently, the contribution of other structural proteins emerged (e.g., MyBP-C, titin), opening to subfilament resolution. In this context, pioneering single molecule studies measured distinct turnover rates along the thick filament regions, and suggest even more complex forms of cooperation among molecules (Nelson et al. 2020).

Many of the structural outcomes evaluated here report changes in myosin orientation or mass distribution with respect to the thick filament axis, while the ones reaching nearly atomic resolution (cryo-EM) are in stabilized systems that, despite the use of mavacamten, still highlight a relevant level of mobility (CrD in Dutta et al.) (Dutta et al. 2023). If certainly the nucleotide hydrolysis events measured by ATPases and mantATP chasing require some level of structural rearrangement, they could happen within the nucleotide binding site and might not involve the whole myosin globular domain. In this regard, the strong coupling of structural rearrangement with nucleotide hydrolysis that was firstly hypothesized could be due to the historical inheritance of early power stroke studies, in which the movements of motor components are tightly forced toward force production. .In resting myosin, the lack of actin contact may provide the flexibility required for nucleotide turnover and ADP release, without necessitating a change in the motor's overall orientation.

**Fig. 2:**
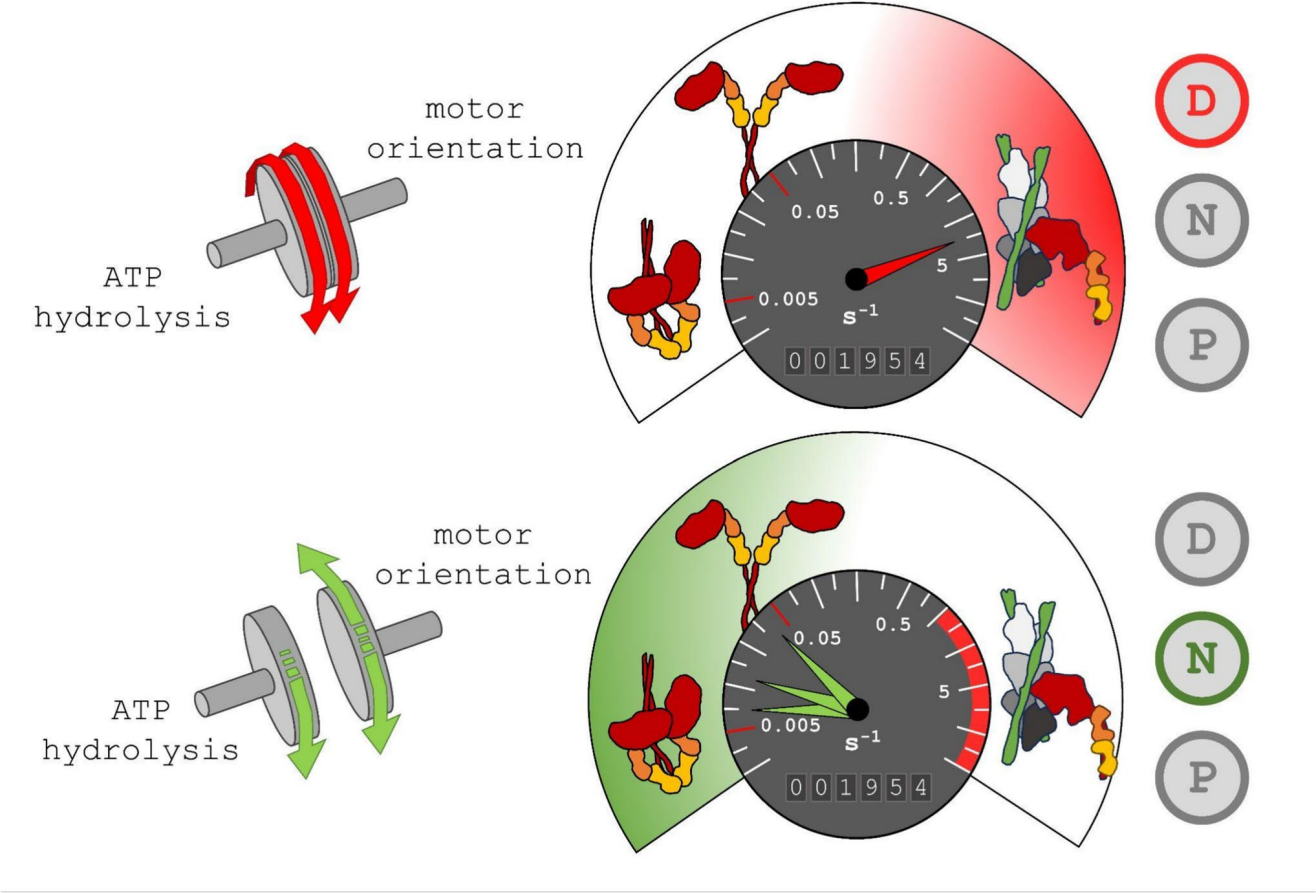
Proposed model of a “molecular clutch” as a variable coupling among myosin orientation and ATP hydrolysis. During muscle contraction, in drive mode (D) the clutch is closed and motor orientation is strongly tethered to nucleotide exchange rate, while in the resting state the clutch is not engaged (neutral, N) or even switched off (P), and ATP hydrolysis and motor orientation are uncoupled. The multiple ATPase rate indicators refer to the different degrees of coupling. The analogy myosin motor-car motor has been previously suggested by Roger Cooke (Cooke 2011) and reprised by Nag and Trivedi 2021 (Nag and Trivedi 2021)

To conclude, we propose the idea of a “molecular clutch” in which the structural rearrangement of the motor and the nucleotide hydrolysis can experience various degrees of coupling (Fig. 2). If, during contraction, actin availability drives the clutch to a tight friction to power the working stroke, in resting conditions the two mechanisms are loosely tethered. In this scenario, molecules modulating myosin resting states could alter the connection between the structure and biochemistry of the head, impacting differently on contractility and energy consumption.

Since its identification by Roger Cooke, the concept of myosin resting states has opened an entirely new field in muscle research—a mechanism grounded in fundamental myology that now extends its influence into translational and clinical domains. Most of the studies discussed here rely on ex vivo preparations that, while rigorous, offer only limited insight into how myosin heads behave in a beating heart or a contracting muscle. What is the in vivo balance between the SRX and DRX states? How does it vary among tissues and from health to disease? With exercise? What is the metabolic cost of destabilized myosin heads at the organismal level? These remain some of the key unanswered questions. Shedding light on these topics would undoubtedly lay the groundwork for new chapters in the physiology textbooks of the future.

## Authors’ commentary

Like many readers, I have been to the famous Alpbach Molecular Motors Meetings multiple times. My personal first was in 2013, when Carlo Reggiani introduced me to Roger. I clearly remember, as a student, the feeling of walking among giants. At that time, I would describe the scientific atmosphere around the super relaxed state as somehow between cold and suspicious. Roger was totally untouched by these feelings as he said, “It’s going to grow.” Years went by, I joined Roger's lab during my PhD, and a couple of Alpbachs later (yes, this is our way of measuring time), it was late evening and Roger and I were strolling the poster session. One by one, many and many more posters were evaluating the SRX, in cardiac tissue, in skeletal muscle. I remember his confident yet relaxed look when he said, “I told you.”.

In this short note, I would like to express my gratitude to Roger as he taught me a lot about science, and a lot about life. LN

## Data Availability

No datasets were generated or analyzed during the current study.
